# Governing heatwaves in Europe: comparing health policy and practices to better understand roles, responsibilities and collaboration

**DOI:** 10.1186/s12961-020-00645-2

**Published:** 2021-02-15

**Authors:** Kirsten Vanderplanken, Peter van den Hazel, Michael Marx, Ahmad Zia Shams, Debarati Guha-Sapir, Joris Adriaan Frank van Loenhout

**Affiliations:** 1 Centre for Research On the Epidemiology of Disasters (CRED), Institute of Health and Society (IRSS), Université Catholique de Louvain, Clos Chapelle-Aux-Champs 30, 1200 Woluwé-Saint-Lambert, Brussels, Belgium; 2 International Network On Children’s Health, Environment and Safety (INCHES), Kastanjelaan 5, 6955AM Ellecom, The Netherlands; 3 Evaplan at the University Hospital, Heidelberg, Ringstr.19b, 69115 Heidelberg, Germany

**Keywords:** Governance, National heat health action plan, Heatwave, Emergency management

## Abstract

**Background:**

The expectation that climate change will further exacerbate extreme weather events such as heatwaves is of primary concern to policymakers and scientists. Effective governance is fundamental to preparedness for and response to such threats. This paper explores the governance structures of European heat health action plans and provides insights into key stakeholders, roles, responsibilities and collaboration.

**Methods:**

This was a two-phase qualitative study, in which we complemented a desk review of 15 European national heat health action plans (NHHAPs) with, after obtaining informed consent, 68 interviews in nine countries with key informants involved in the development, implementation and/or evaluation of these NHHAPs. A thematic analysis was used to analyze the NHHAPs inductively. This analysis focused on three themes: identifying key stakeholders, defining and assigning roles and collaboration among stakeholders. The iteratively created codebook was then applied to the analysis of the key informant interviews. All analyses were done using NVivo 10 qualitative analysis software.

**Results:**

The majority of the NHHAPs have governance as one of their main objectives, to support the coordination of actions and collaboration among involved stakeholders. There are, however, significant differences between plan and practice. On the basis of the available data, we have little insight into the process of stakeholder identification, but we do find that most countries involve the same types of stakeholders. Roles are mainly defined and assigned in relation to the alert levels of the warning system, causing other role aspects and other roles to be vague and ambiguous. Collaboration is key to many NHHAP elements and is mainly experienced positively, though improvements and new collaborations are considered.

**Conclusions:**

Our findings show a need for a more deliberate and structured approach to governance in the context of NHHAPs. A cross-sectoral approach to the identification of key stakeholders can facilitate a broader preparedness and response to heatwaves. Roles and responsibilities of stakeholders should be defined and assigned more clearly to avoid confusion and to improve effective implementation. To this extent, we identify and describe seven key roles and potential stakeholders to which these roles are usually assigned. Finally, also collaboration among stakeholders can benefit from a cross-sectoral approach, but also formal structures can be beneficial.

## Background

The severe heatwave that occurred in 2003 resulted in an excess mortality that exceeded 70 000 in Europe [1, 2]. This mortality crisis in combination with the anticipation of global warming exacerbating the frequency, duration and intensity of future heatwaves prompted many European countries to develop national heat health action plans (NHHAPs) to protect public health [3–5]. While the development of NHHAPs has likely changed the heat–mortality relationship, recent heatwaves continue to have devastating impacts on public health [6–8]. For example, the record-shattering heatwaves of 2019 caused over 2 500 excess deaths in Belgium, France and the Netherlands [2, 7]. The continued and increasing threat of heatwaves underlines the relevance and importance of NHHAPs and adapting these on the basis of lessons learned.

Despite the widespread adoption of NHHAPs, no two countries’ strategies are the same and multiple strategies can exist even within countries. Nevertheless, there are international guidelines available that can be of help to public health officials (e.g. [9, 10]). In addition, working groups such as the Working Group on Health in Climate Change established by the European Environment and Health Task Force in 2012 aim to foster international dialogue and learning in developing and implementing national action plans.

NHHAPs usually include a set of strategies: (i) an early warning system based on meteorological and epidemiological parameters to forecast heat events and their health impact; (ii) specific actions to prevent negative health effects of heat targeted at the general public or specific vulnerable groups; (iii) a communication plan to raise awareness and improve preparedness in stakeholders and citizens; and (iv) governance structures to coordinate actions and collaborations [3, 4, 10, 11]. Within this paper, we focus on the fourth element as we intend to contribute to understanding governance structures in the context of NHHAPs in Europe. Specifically, we aim to provide insight into which stakeholders are involved in NHHAPs, how roles are defined and assigned, and how stakeholders collaborate.

In recent decades, there has been a tendency to decentralize emergency management and to assign stakeholders from different levels responsibilities that were formerly taken by the national government [12]. This trend also emerges in the prevention and management of heatwave risks. NHHAPs are developed at the national level, but the implementation relies heavily on regional and local stakeholders who are presumed to be more familiar with local needs and thus better able to respond. Moreover, responsibilities are not limited to public stakeholders, but also non-profit and for-profit agencies, as well as citizens, can be involved. Today, this practice is known as “governance”, that is the multitude of inter-sector and inter-governmental structures and processes aimed at collective decision-making [13–15]. More specifically, the term “risk governance” can be used to describe the application of governance principles to structures and processes for identifying, assessing, managing and communicating risks, such as those related to heatwaves [15, 16]. There is a need to better understand how public and private actors can govern heatwave risks more effectively, especially since traditional power and authority structures have been proven inadequate in this context (hence the redistribution of responsibilities described above) [16].

For governance to be effective, it is key to develop governance structures that define how roles and responsibilities are assigned and distributed among key stakeholders (i.e. the sets of tasks assigned to a stakeholder within the context of a NHHAP), and how workable collaborative relations can be developed [17]. These collaborative relations involve working together to achieve the collective goals of the NHHAP, sharing authority between stakeholders to enable tailoring to local conditions, and exchanging resources. Since the development of a NHHAP takes place at the national level, roles and responsibilities are assigned in a top-down manner to regional and local stakeholders. This practice, however, is not without its difficulties. Previous evaluations of NHHAPs have often found that there is a need to define roles and responsibilities more clearly and to improve collaboration among stakeholders [10]. Several studies show that local stakeholders, especially those in health and social services, are not always sufficiently aware of the NHHAP or their role in it [18–20], which undermines the success of their involvement [21]. Moreover, when NHHAPs do include role assignments and descriptions, they are perceived to be insufficiently detailed, causing confusion and hindering uptake of responsibilities [18, 19]. Additionally, without legal enforcement, some stakeholders can decide not to perform the role assigned to them [18]. By defining and assigning clear roles, a NHHAP can guide relevant stakeholders in addressing heat health risks, support implementation and create a structure of coordination [10, 22]. It is important for local and regional efforts to have a strong foundation at the national level, and clearly assigned roles and authority are part of this [22]. Role clarity can help to prevent overlapping functions and unfunded mandates, and helps to avoid certain tasks being neglected or not performed because no stakeholder is willing or obliged to take on the responsibility [23]. Nevertheless, stakeholder involvement not only depends on the relationship between the national and lower levels. Local stakeholders can only be involved when they have the capacity, knowledge, motivation, network and procedural capacity to do so [21]. Moreover, input from local stakeholders should feed back into the development and evaluation of a NHHAP [18]. Without clear role divisions and coordination at all levels, there is a risk that the implementation of NHHAPs cannot effectively or efficiently manage heat health risks.

The present paper describes governance structures in current NHHAPs and compares these to the reality in the field with specific attention to the identification of key stakeholders, the process of defining and assigning roles and collaboration, and descriptions of key roles. Our results provide insights into the governance structures used in European NHHAPs and their strengths and weaknesses. These insights can contribute to the development of new NHHAPs and the evaluation and adaptation of existing ones, in Europe and beyond.

## Materials and methods

The data collection and analysis occurred in two consecutive steps. First, a desk review of NHHAPs in European countries was conducted, which was then followed by key stakeholder interviews. Below, we first discuss the data collection process for each data source, and then the analysis.

### Data collection

#### Desk review of NHHAPs

The desk review was conducted between April and July 2019 and focused on identifying NHHAPs in European countries. We started by identifying NHHAPs in review articles (e.g. [24–26]). Next, for those European countries that were not mentioned in these articles, we conducted online searches using the same keywords for each country (country name + national heatwave plan; country name + heat alert; country name + national heat health warning system; country name + plan + heat + health). The online searches were conducted in English, and when necessary complemented with searches in the countries’ main languages using Google Translate. When the online searches did not yield results, we searched the websites of public health authorities and meteorological institutes. By using the Google Translate plug-in we were able to understand the content of websites that were not available in English or another language covered by the research team. A final step was to reach out to public health authorities or meteorological agencies, and to researchers who had published about a certain country’s NHHAP. In total, we collected NHHAPs from 15 European countries (Table 1). A study by Bittner et al. [27] described additional NHHAPs of Hungary, Monaco, Moldova, Romania and Serbia, but we were unable to retrieve these plans. Also Cyprus may have developed a NHHAP following an EU LIFE project [28], which we could also not retrieve. Further, we identified other HHAPs that did not match our criteria, such as regional plans or plans not approved by national authorities or agencies.

**Table 1:** Overview of retrieved NHHAPs and key informant interviews

Country	NHHAP (title translated)	Key informant interviews		
		Respondents	Format	Date
Austria	Heat protection plan [36]			
Belgium	Federal plan for heat and ozone peaks [37]	1. Public health agency 1 2. Cross-government agency 3. Public health agency 2 4. Public health agency 3 5. Meteorological agency 6. Care provider 7. NGO	Face to face Face to face Face to face Face to face Face to face Face to face Face to face	18/07/2019 10/07/2019 10/07/2019 10/07/2019 10/07/2019 22/10/2019 22/10/2019
Finland	Health care cold and heat guide. Model for use by regional actors 2011–2012 [38]			
France	National heatwave plan [39]	8. Care provider 1 9. Social institution 10. Public health agency 1 11. Care provider 2 12. Meteorological agency 13. Public health agency 2 14. Local authority 15. NGO	Face to face Face to face Face to face Face to face Face to face Face to face Phone Email	17/06/2019 17/06/2019 19/06/2019 18/06/2019 19/06/2019 18/06/2019 02/07/2019 20/08/2019
Germany	1. Climate change and health—information on health effects of summer heat and heatwaves, and tips for preventive health protection [40] 2. Recommendations for action—heat action plans to protect human health [41]	16. Social institution 17. Care provider 18. National agency 19. Ministry 20. Emergency services 21. Meteorological agency 22. Public health agency	Face to face Face to face Face to face Face to face Face to face Face to face Email	07/08/2019 07/08/2019 05/08/2019 24/07/2019 08/08/2019 19/07/2019 03/09/2019
Italy	National prevention plan for the effects of heat on health [42]			
Lithuania	Order on national public health and heat prevention [43]			
Luxembourg	Action plan in case of great heat [44]			
North Macedonia	Heat-health action plan. To prevent the consequences of heatwaves on the health of the population in the former Yugoslav Republic of Macedonia [45]	23. Meteorological agency 24. Public health agency 1 25. Public health agency 2 26. NGO 27. Ministry 28. Crisis agency 29. Emergency services 30. Local authority	Email Face to face Face to face Face to face Skype Email Phone Phone	04/09/2019 20/08/2019 20/08/2019 20/08/2019 10/09/2019 03/09/2019 17/09/2019 24/09/2019
Netherlands	National heat plan [46]	31. Public health agency 32. Ministry 33. NGO 34. Meteorological agency 35. National agency 36. Local agency	Face to face Face to face Face to face Face to face Face to face Face to face	23/07/2019 13/08/2019 19/07/2019 02/09/2019 17/07/2019 16/08/2019
Portugal	Contingency plan for extreme adverse temperatures—heat module [47]	37. National agency 38. Regional authority 39. Care provider 40. Ministry 41. Meteorological agency 42. Local agency 1 43. Local agency 2	Face to face Face to face Face to face Face to face Face to face Face to face Face to face	07/08/2019 05/08/2019 07/08/2019 05/08/2019 07/08/2019 08/08/2019 08/08/2019
Spain	National plan for preventive actions for the effects of excess temperatures on health [48]	44. Care provider 45. Meteorological agency 46. Regional authority 47. Ministry 1 48. Local authority 49. Ministry 2 50. Local agency 51. Social institution	Face to face Face to face Face to face Face to face Email Face to face Face to face Face to face	28/06/2019 25/06/2019 26/06/2019 28/06/2019 22/07/2019 27/06/2019 27/06/2019 27/06/2019
Sweden	Managing health effects of heatwaves—guidance to action plans [49]			
Switzerland	Epidemiology and public health—heatwave measures toolbox [50]	52. Public health agency 53. Meteorological agency 54. Regional authority 1 55. Care provider 1 56. Ministry 57. Regional authority 2 58. Care provider 2	Skype Phone Skype Phone Email Phone Email	04/09/2019 04/09/2019 17/09/2019 19/09/2019 02/10/2019 09/10/2019 15/10/2019
UK	Heatwave plan for England—protecting health and reducing harm from severe heat and heatwaves [51]	59. Local agency 60. NGO 61. Meteorological agency 62. Public health agency 1 63. Community group 64. Public health agency 2 65. Emergency services 66. Public health agency 3 67. Public health agency 3 68. Public health agency 3	Face to face Face to face Face to face Face to face Face to face Face to face Face to face Face to face Phone Face to face	01/07/2019 29/07/2019 29/07/2019 29/07/2019 30/07/2019 03/07/2019 29/07/2019 29/07/2019 29/07/2019 29/07/2019

Following the example of Bittner et al. [27], we included a plan as a NHHAP when (i) it was issued on the national level by a national authority or agency, and (ii) when the response to heat and heatwaves was a main topic. For practical purposes, we could only include NHHAPs that were available in English, Dutch or French, or could be reliably translated to English using Google Translator. Some of the included NHHAPs were older (e.g. the French plan was first developed in 2004) and have been evaluated and updated multiple times, whereas others were developed more recently (e.g. the Lithuanian plan was developed in 2015) and have not yet been evaluated.

#### Key informant interviews

Following the desk review, we conducted 68 key informant interviews (Table 1) to gain insights into the implementation of NHHAPs in the field, to identify strengths and weaknesses, and to find best practices. The interviews were conducted in nine out of the 15 countries for which we were able to retrieve a NHHAP. Our selection aimed to capture the wide variation in NHHAPs: we included NHHAPs from different parts of Europe and with different characteristics, such as the age of a NHHAP (based on the year in which a first version was published) and its legal context (e.g. NHHAPs of which parts have been institutionalized in laws and non-committal NHHAPs).

In each of the selected countries, we conducted interviews with 6–10 key informants (see Table 2 for details). Stakeholders (e.g. policymakers, public institutes, care providers) were eligible for participation when the analysis of the NHHAPs indicated that they have a role in their country’s NHHAP. We identified key informants in three ways: (i) their name or that of their organization was mentioned in the plan (e.g. the authors, the Red Cross); (ii) the type of organization they represent was mentioned in the plan (e.g. hospitals, regional authority); and (iii) through snowballing. For the first two strategies, we conducted online searches to retrieve contact information if this was not included in the NHHAP. For the third strategy we asked the referring party to provide the contact information. All key informants were first approached via email, which was followed-up with a telephone call in case of non-response. We selected stakeholders who perform different roles within each country, and with activities on different levels (municipal, regional or national). For those stakeholders who are active on other levels than the national, we added the criterion that they needed to be based in the capital or region in which the capital is located. As capitals are usually larger cities, we believed that they were more likely to be active in the field of heatwave preparedness, and it also allowed us to limit travel time. In Germany and Switzerland, an additional city besides the capital was included (respectively Bonn and Lausanne), as in both countries stakeholders in the capital were less involved in heatwave preparedness compared to the second city.

**Table 2:**
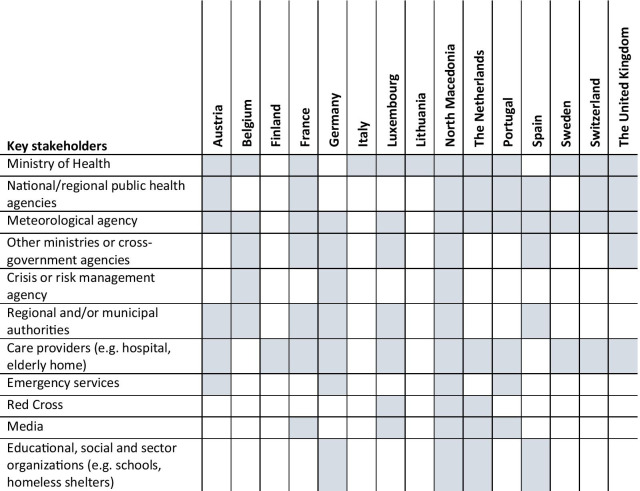
Overview of key stakeholders to involve in a NHHAP

The interviews were conducted by six interviewers from three partner organizations (including the first author). All interviewers were trained and supported by the first author. We developed an interview protocol to further ensure comparability across countries and interviewers, which included a semi-structured interview guide. The questions in the interview guide were informed by the analysis of the NHHAPs, and suited the exploratory nature of the interviews while still ensuring comparability. Specifically, we included questions on the following themes: (i) role of the organization within the NHHAP; (ii) existence of and details on organizational HHAP; (iii) responsibilities of the organization in case of a heatwave; (iv) collaboration with other stakeholders; and (v) evaluation of the (N)HHAP. The protocol also included consent forms (in English and translated to native languages) and we obtained informed consent from all respondents prior to the start of the interviews. The interviews were conducted face to face or, if this was not possible, via Skype, phone or—as a last resort and in very few cases—the interview was completed via email. The interviews lasted between 20 and 90 min. All interviews were conducted in either the respondent’s native language (Dutch, English, French, Portuguese, Spanish, German) or English. The interviews were audio recorded, transcribed literally and—if necessary—translated to English by the interviewer. Transcripts were reviewed by the first author, and were revised or clarified in collaboration with the interviewer.

### Data analysis

Both types of data were analyzed inductively using NVivo 10 qualitative data analysis software. By using an inductive approach, we were able to (i) condense the extensive amount of data collected, and (ii) develop insights into governance structures used in NHHAPs through the development of themes from the raw data (i.e. thematic analysis) [29]. The codebook was iteratively created through the sequential analysis of the NHHAPs and interviews.

The data analysis started as soon as the first NHHAPs were retrieved. First, we conducted a descriptive analysis of the NHHAPs to identify key characteristics (e.g. country, date of creation, authors). Next, we used axial coding to organize the content of the NHHAPs in thematic categories that were directly derived from the data. Examples of thematic categories include “roles”, “stakeholders” and “organizational scheme”. By reading and rereading the NHHAPs, we identified, examined and categorized all data relevant to each thematic category in a process of constant comparison [30]. The analysis of the NHHAPs served as a basis for the key informant interviews and was especially important for the selection of key informants, which was based on the role(s) they perform within a NHHAP, and for the development of the interview guide.

The second phase of the analysis focused on the interviews. All interview transcripts were imported in the same NVivo file as the NHHAPs. This allowed us to use the same codebook and method of analysis. Again, we first conducted a descriptive analysis to identify characteristics of the key informants (e.g. country, interviewer, organization), which was followed with a thematic analysis using axial coding. When new thematic categories emerged, they were iteratively added to the codebook.

This sequential analysis approach allowed us to compare between the two data sources and to compile information across countries in an easy and effective way.

## Results

Following the desk review, we found that a NHHAP generally targets up to four objectives that align with the set of strategies identified in the literature [3, 4, 10, 11]: (i) to forecast heat events in a timely manner, (ii) to prevent negative health effects of heat, (iii) to raise awareness on the health risks of heat among stakeholders and the general public, and (iv) to coordinate actions and collaboration among involved stakeholders. In this paper, we focus on the fourth objective, which concerns governance. Specifically, we identify three aspects of governance structure that a NHHAP needs to address: (i) identifying key stakeholders; (ii) defining and assigning roles; and (iii) collaboration among organizations. Below, we discuss our results for each aspect. By separately discussing results from the desk review of NHHAPs and key informant interviews, we highlight the differences between plan and field realities.

### Identifying Key Stakeholders

#### NHHAPs

Because the identification of stakeholders usually takes place before the NHHAP is written, this process is generally not described within the plans. The German and Swiss documents provide some insights and include a general description of which stakeholders to involve, largely based on the WHO recommendations [10, 27]. On the basis of the author lists in the NHHAPs, we found that usually the meteorological agency, Ministry of Health and public health agencies play a key role in the development and implementation of a NHHAP. In addition, other important stakeholders in most NHHAPs are regional and municipal authorities, care providers (e.g. general practitioners, elderly homes), emergency services, social services, schools and NGOs (e.g. the Red Cross).

#### Interviews

The interviews highlight the importance of involving stakeholders from other sectors besides health (e.g. schools, trade unions) to ensure relevance and improve uptake.

“I think that over the last ten years we have built up good cooperation and networks, both at the national level and at the state level or even between the state level and the municipalities.[…] What is needed is a central body to manage such an action plan and then work together with the various users. There is the administration of various official institutions, but also aid organizations, hospitals, medical professions, pharmacists, the Red Cross, and so on and so forth… A wide range, schools, kindergartens and this network, the local level.” (Germany, national agency).

However, respondents recognize it may be difficult to identify and engage stakeholders from other sectors, as there are often no pre-existing relations or prior knowledge of which stakeholders are relevant to involve. Therefore, key stakeholders involved in the NHHAP identified through the interviews are the same as those identified on the basis of the desk review.

### Defining and assigning roles

#### The process

##### NHHAPs

Roles are mainly defined and assigned in relation to the alert levels of the warning system (e.g. in North Macedonia, alert levels 1–3 are activated by the meteorological agency and a separate commission is responsible for activating level 4). Some NHHAPs also include overarching roles, such as those related to communication or evaluation. As the Swiss and German NHHAPs were seemingly written without previously identifying and involving stakeholders, they only provide general role descriptions without assigning these to specific stakeholders and alert levels. Finally, some NHHAPs enforce certain roles by law, e.g. the French, Portuguese, Spanish and British NHHAPs.

##### Interviews

While most respondents believe their organization’s roles are clearly defined in the plan, they also identify several issues. First, there is a lack of detailed information on how to implement roles in practice. Consequently, some stakeholders are assigned roles which they do not know how to perform or where to get the required resources from. In addition, this leads stakeholders to believe the role does not concern them and as such they may avert the responsibility. Second, some stakeholders lack the autonomy to execute certain roles when these deviate from their regular organizational tasks. This issue particularly concerns care providers and social and educational institutions.

“So, the plan is one of the many activities that we carry out […] Sometimes we have difficulty in responding to what is recommended in the plan and to perform our role. We cannot guarantee it, because we do not have the autonomy to do it. But we try to alert people that infrastructure, for example, is not always prepared to respond to the extent we would like.” (Portugal, local agency 1).

#### Key roles

Each stakeholder can perform multiple roles within a NHHAP, and each role can in turn be performed by multiple stakeholders. Below, we describe the responsibilities related to the seven roles we have identified in the desk review. Through the analysis of the interviews, in which we included specific questions on role responsibilities, we can explore the extent to which actual responsibilities equal those in the NHHAPs. In addition, we provide an overview of the types of stakeholders to which these seven roles are usually assigned (Table 3).

**Table 3:** Key roles and the stakeholders assigned to them

Role	Stakeholders	
	NHHAPs	Interviews
Author	Multiple authors collaborate such as ministry of health and care providers One stakeholder takes lead (usually ministry of health)	Multiple authors collaborate such as ministry of health, public health and environmental agencies, research institutes and care providers Number of authors can grow over time Stakeholders from various sectors can contribute expertise
Activator	National/regional authority, meteorological agency or public health agency Possible to assign different activators for different alert levels	National or regional level stakeholder (e.g. meteorological agency, ministry of health, public health agency, regional authority)
Coordinator	Multiple coordinators on different levels possible, e.g. ministry of health at national level, regional or municipal authorities, care providers and social institutions at lower levels One coordinator takes lead	Multiple coordinators for different levels recommended, e.g. ministry of health at national level and regional/municipal authorities at lower levels
Evaluator	Not always clearly assigned Seemingly assigned to 1 type of stakeholder such as ministry of health, public health agencies, regional or municipal authorities, care providers	≥ 1 national level stakeholder such as ministry of health or public health agencies
Implementer	Stakeholders at all levels, such as ministry of health, public health agencies, care providers, educational and social institutions, NGOs and community groups	Municipal stakeholders such as municipal authorities, public health agencies, care providers, social institutions, NGOs
Informer	Internal informers are national or regional level stakeholders (e.g. ministry of health, meteorological agency, public health agencies) External informers are stakeholders at all levels (internal informers plus e.g. care providers, educational and social institutions, media, NGOs)	A national or regional level stakeholder (e.g. ministry of health, public health agency, crisis agency) develops and supplies information materials Other stakeholders diffuse the information, such as media, meteorological agency, public health agencies, municipal authorities, NGOs
Monitor	Stakeholders with access to relevant data such as meteorological agency, ministry of health, public health agencies and crisis agencies	Stakeholders with access to relevant data such as meteorological agency, public health agencies, research institutes, authorities

Both the information in the table and below is summarized for the purpose of this paper. Hence, the provided information might not equally apply to all countries. A more detailed overview can be obtained from the authors.

- Author
  *NHHAPs* An author is responsible for developing and writing the plan.
  *Interviews* According to the respondents, the role of author is considered to be a continuous responsibility, with the NHHAP being iteratively completed and adapted on the basis of experiences and lessons learned.
- Activator
  *NHHAPs* An activator is responsible for activating the plan and/or warning system. This includes communicating the activation to internal and external stakeholders and the public (usually at least 1 day before the occurrence or aggravation of the heat event) and activate plan implementation by all involved stakeholders (e.g. implementation of measures, activation of crisis cell).
  *Interviews* The interviews confirm the responsibility of activators to activate the plan and to communicate this to internal and external stakeholders. The latter is considered to be of particular importance. In addition, activators are also considered to be responsible for activating plan implementation, and are required to collaborate and exchange information with the monitor(s) and coordinator(s). The respondents point out three issues: activators are unsure how to adequately communicate activation to all external stakeholders and the public; they often cannot ensure the activation of NHHAP implementation on all levels; and they have difficulty ensuring activation and implementation of measures during prolonged periods of activation.
- Coordinator
  *NHHAPs* A coordinator is responsible for coordinating implementation and cooperation. The responsibilities are often fragmented across levels, sectors and/or tasks, indicating a need for multiple coordinators as well as a general coordinator.
  *Interviews* The respondents confirm that coordinators are responsible for coordinating efforts within and across organizations. Owing to the scale and diversity of organizations that need to be coordinated, there is a need for multiple coordinators who have good skills and access to sufficient resources. However, respondents indicate a lack of good coordination, particularly at subnational levels.
- Monitor
  *NHHAPs* A monitor is responsible for monitoring meteorological and/or epidemiological parameter(s). This includes anticipating when critical values may be reached and informing relevant stakeholders in a timely manner. In addition, monitors can be asked to provide advice on the definition of parameters and thresholds.
  *Interviews* The respondents fully confirm the responsibilities of monitors as described in the NHHAPs.
- Informer
  *NHHAPs* An informer is responsible for diffusing information. Internal informers are responsible for informing authorities and specific stakeholders identified in the plan about parameter values and activation or escalation of an alert level. External informers are responsible for informing stakeholders not included in the internal information loop and the general public, including vulnerable groups, about the occurrence of a heatwave, to raise awareness about heat health risks and to provide information on protective measures. At the national level, the role of informer can also include developing information material for internal and/or external diffusion.
  *Interviews* On the basis of the interviews, the role of informers is to diffuse information and ensure that preventive action will be taken. According to the respondents, the NHHAPs do not contain sufficient information on the specific responsibilities of informers and how to realize these (e.g. who informs whom, how to identify and reach specific target groups).
- Implementer
  *NHHAPs* An implementer is responsible for implementing the measures described in the NHHAP. Most stakeholders involved in the NHHAP are implementers in some way, so responsibilities greatly differ between stakeholders and are usually related to the activated alert level.
  *Interviews* The specific responsibilities of implementers are often unclear to respondents, particularly for subnational stakeholder. Two explanations are given: the NHHAPs do not describe the responsibilities for subnational implementers in sufficient detail, and the information on responsibilities does not adequately reach the implementers. As a result, they may not be able to perform the role adequately or they may deviate from the NHHAP (or its intentions) and adapt the role to local and organizational circumstances.
- Evaluator
  *NHHAPs* An evaluator is responsible for evaluating the national heatwave plan. This includes evaluating the effectiveness and relevance of implemented measures, assessing the accuracy and impact of parameters and threshold values, identifying difficulties and lessons learned and updating the NHHAP. Evaluation can be done before and/or after summer, and sometimes also during the occurrence of a heatwave.
  *Interviews* Since the responsibilities of evaluators and the process of evaluation are often not described in the NHHAP (e.g. which indicators to use, where to get data and feedback on implemented measures), this role is rather informal. Consequentially, it is not performed systematically, either in timing or in process. Nevertheless, when the role is taken on, evaluations do feed back into NHHAP updates.

###  Collaboration Among Stakeholders

#### NHHAPs

Within the NHHAPs, collaboration is mostly mentioned regarding the development of a NHHAP, the warning system and its parameters, communication, information exchange and coordination. The NHHAPs discuss collaboration between institutions, between national, regional and local levels, and between sectors. Some plans aim to formalize these relations to make the collaboration more effective, for instance with collaboration agreements (e.g. the Spanish NHHAP).

#### Interviews

The interviews describe existing collaborations between stakeholders from different institutions (governmental and non-governmental) and between stakeholders from the same and different levels (i.e. national, regional, municipal, organizational). Stakeholders report to collaborate with others that have access to skills, information, resources or connections they are lacking.

“The levels [i.e. parameter thresholds] are defined by [the public health agency] and [the meteorological agency] jointly.[…] So, it's really a work in common between us. Because [the public health agency] works on mortality issues and we are working on the physical issues of the atmosphere. And it is in crossing both domains, that we can define the thresholds.” (France, meteorological agency).

Although the respondents report to be satisfied with the quality of current collaborations, some issues are mentioned. Specifically, there is a need for more inter-sectoral collaboration, for more and better collaboration with(in) the health sector, for collaborative instead of top-down relations with government stakeholders, and for structures that make collaboration easier and lessen administrative difficulties. Finally, respondents mention that they currently do not engage in structural cross-country collaborations, though they would like to invest in this in the future.

## Discussion

Under current climate predictions, heatwaves are expected to occur more frequently in the future with increasing intensity and duration. To prepare for and respond to heatwaves and to minimize their health impact, many countries have developed NHHAPs. Previous studies [10, 18–20] have found that the governance aspect of NHHAPs needs to be improved. Public health officials are not always sure how to develop an effective governance approach. As a result, we find variation in the extent to which governance is actually considered and described within NHHAPs. This article discusses currently used governance approaches in European NHHAPs, with the aim of supporting the development of new and evaluation of existing NHHAPs. We provide insights into how key stakeholders are identified, how key roles are defined and assigned, and how collaboration currently occurs. These findings do not need to be applied uniformly in all settings, as national, regional and local conditions vary and need to be considered.

Since NHHAPs are national documents, governance at the national level is a first priority and usually described in most detail. Yet, preparedness for and response to heatwaves are mostly regional or municipal responsibilities and governance on these levels is as important as it is on the national level. Many NHHAPs do consider the regional level in some way, either by describing regional governance structures (e.g. the French NHHAP) or by pointing out the need for or existence of regional HHAPs (e.g. the German NHHAP). The municipal level is rarely considered within NHHAPs. From the interviews we learn that NHHAPs that include multilevel governance can foster timely actions and homogeneity in heatwave management, which helps to avoid confusion and duplicate efforts between governance levels.

### Identifying Key Stakeholders

The identification of key stakeholders ideally takes place before or during the development of a NHHAP. This allows for the involvement of stakeholders in an early stage, which can foster engagement, uptake and overall effectiveness [18]. However, there are some exceptions in which NHHAPs do not identify key stakeholders or not in very much detail. We see this, for example, in NHHAPs that are developed as non-committal guidelines (e.g. the German and Swiss NHHAPs). As a result, the implementation and uptake of these plans is much lower and more dependent upon the motivation of and prioritization by their intended users (often regional authorities).

Further, it is recommended that NHHAPs are not limited to the health sector as heatwave vulnerability also includes environmental, social and technical dimensions [31]. For example, stakeholders such as schools, trade unions and sports clubs should be identified and actively involved. Since the networks of currently identified stakeholders often do not reach these other sectors, this requires additional and active consideration. By taking a cross-sectoral approach early on, for example by commissioning a stakeholder with a cross-sectoral network to lead the identification (and involvement) process, this issue could be mitigated.

### Defining and assigning roles

#### The process

Within the studied NHHAPs, roles are mainly defined and assigned in relation to the alert levels of the warning system. Specifically, the roles of activator and informer are more clearly described and more detailed, whereas descriptions on the roles of coordinator, evaluator, implementer and monitor are more general. Some NHHAPs also define and assign overarching roles. Overall, the focus on roles relating to alert levels does indicate a focus on response, which implies that mitigation, preparedness and evaluation remain un(der)addressed.

Further, we find that roles in NHHAPs are usually defined and assigned in a top-down manner. While some top-down coordination is required and can help to encourage all stakeholders to achieve the desired objectives, it needs to be combined with consultation, motivation and cooperation of subnational stakeholders [4, 12]. By involving stakeholders in the definition and assignment of roles, better governance can be achieved [10]. Such a hybrid governance approach may also succeed in mitigating two other issues identified in this study as well as in previous work. First, all stakeholders should be aware of and accept their role in the NHHAP [27, 32]. Second, all roles should be clearly defined and described in order for stakeholders to know what exactly is expected of them [18, 19]. Through early involvement and collaborative decision-making processes, it can be ensured that stakeholders are familiar with the NHHAP and will perform the role(s) assigned to them. In addition, it allows stakeholders to weigh on decisions about rules and procedures, provide input on role definitions and assignments, address unclarities, and discuss organizational constraints or requirements, such as resources, that may affect role performance. However, within the context of NHHAPs, this decentralization and reorganization of governance structures does not mean the state has become a less relevant actor or that all responsibility is placed with individual citizens.

#### Key roles

Our analysis reveals inconsistencies between what is prescribed by the NHHAPs and actual practices: role definitions in the NHHAPs deviate from those given by respondents, and some stakeholders take on more or completely different roles than those assigned to them by the NHHAP. Often, these differences seem to arise where respondents indicate that the NHHAP does not provide sufficiently detailed role definitions. Instead, stakeholders will use their own interpretations on how to realize and implement certain actions. This can lead to heterogeneity in the actions taken, confusion and disagreement about who should take responsibility [19]. It is thus paramount that a NHHAP includes clear definitions of roles and responsibilities. This will facilitate role performance (e.g. by providing specific information on how to target different audiences) and ensure that roles are assigned to those stakeholders who have the skills and resources to perform the roles.

#### Box 1. Key roles: definition, responsibilities and/or required skills and resources

**Table:** 

Author	Stakeholder responsible for developing and writing the plan in a continuous, iterative process Relevant national and subnational stakeholders from health and other sectors should be involved, but it is recommended that one stakeholder takes the lead
Activator	Stakeholder responsible for activating the plan and/or warning system Responsibilities include decision-making, communicating the activation to internal and external stakeholders as well as the public, activating plan implementation and collaborating with the monitor(s) and coordinator(s) Performing this role requires authority and communication lines with other stakeholders
Coordinator	Stakeholder responsible for inter-organizational coordination of plan implementation and collaboration Performing this role requires authority, an inter-organizational network, coordinating skills and sufficient resources
Evaluator	Stakeholder responsible for evaluating the NHHAP and/or the situation and plan implementation during the occurrence of a heatwave Responsibilities include assessing the effectiveness and relevance of implemented measures, assessing the accuracy and impact of parameters and threshold values, identifying difficulties and lessons learned and updating the NHHAP Performing this role requires a systematic approach and the definition of clear indicators for evaluation
Implementer	Stakeholder responsible for implementing the measures described in the NHHAP (e.g. check on vulnerable people, ensure availability of drinking water) Performing this role requires a clear definition of actions and how to realize them
Informer	Stakeholder responsible for diffusing information to internal and/or external stakeholders and/or the public, preferably in a two-directional communication flow Performing this role requires access to information material, identification of target audiences; connections to internal and/or external stakeholders and communication skills
Monitor	Stakeholder responsible for monitoring parameter(s) Responsibilities include anticipating when critical values may be reached, informing relevant stakeholders and providing advice on the definition of parameters and thresholds

### Collaboration among stakeholders

In general, sufficient collaboration and cooperation are necessary to ensure consistency in the implementation of a NHHAP, to improve uptake among stakeholders and to effectively achieve the objectives [3, 33, 34]. Collaboration allows for the exchange of knowledge and resources and promotes sharing of responsibilities and mutual decision-making. Depending on the purpose of the collaboration or cooperation, different networks may be required (e.g. a central network and a network connecting national stakeholders to local organizations).

We find that while the respondents are satisfied with the current collaborative relations, there is room for improvement. This is consistent with previous work [4, 18–20]. The collaborations we identified are usually limited to an organization’s own network and do not often go beyond the health sector. To promote collaboration in wider networks across sectors, a formal structure (e.g. institutional collaboration platform) can be beneficial [18–20]. Such a structure can make collaboration easier and lessen administrative barriers stakeholders may experience when reaching out to other organizations. In addition, international collaborations with the aim of exchanging knowledge and experiences can contribute to the improvement of NHHAPs and their implementation. Multiple respondents point out that they would benefit from systematic international collaboration or a learning network that allows them to exchange best practices and lessons learned. Since such networks do exist (e.g. the Working Group on Health in Climate Change of the European Environment and Health Task Force), there is a need for increasing familiarity with and access to these networks among stakeholders (especially at lower levels) and improving the dissemination of their efforts.

### Limitations

The methods used for our analysis have some limitations. First, with regards to the desk review of NHHAPs, we may have identified out-of-date NHHAPs (some were updated since the search was conducted) or failed to identify some. Thanks to the contact with stakeholders for the interviews, we were able to minimize the first issue and validate the collected information. Further, we were unable to use some of the identified NHHAPs because they were not available for download or not available in a format or language that could be reliably translated using Google Translate. The use of Google Translate had the additional limitation that some parts of the translation may not be entirely reliable. Despite these limitations, we were able to identify and analyze 15 NHHAPs, which will aid in further informing the development and improvement of NHHAPs in Europe.

Second, concerning the interviews, a possible limitation is that the interviews were conducted by six different interviewers with varying degrees of expertise. However, we believe that the variation in interview approaches was minimized by the interview protocol and guidance provided to the interviewers by the first author. All interviewers were trained to carefully consider the interactions integral to the interview and the possible impact these may have on the outcomes. For instance, interviewers were instructed to utilize positive communication techniques (e.g. listening, being open, knowing when to stay silent).

Another limitation was posed by language as we were dependent on the language skills of the interviewers to conduct interviews in the native language of respondents (or English), and then translate those interviews to English. The potential impact of language was minimized by selecting interviewers whose native language was one of those spoken by the respondents (i.e. Dutch, French, Portuguese) or who were very proficient in that language (i.e. German, English, Spanish). However, particularly for North Macedonia and Switzerland it was not possible to conduct the interviews in the native language of the respondents and some misunderstandings may have occurred as a result of language barriers. Finally, we did not manage to conduct all interviews face to face, but had to resort to telephone, Skype or email in some cases. Since we did not find that those interviews significantly differed from those conducted face to face, we believe that the impact of this is limited.

## Conclusion

Under current climate predictions, heatwaves are expected to occur more frequently in the future with increasing intensity and duration. NHHAPs are used to prepare for and respond to heatwaves and minimize their health impact. However, public health officials are not always aware of the best governance approach to use in a NHHAP, and there is a wider debate among scholars and practitioners about the best approach. Our findings show a need for a broader and more structured approach to governance in the context of NHHAPs: involving stakeholders from multiple sectors beyond health, including stakeholders in governance processes such as defining and assigning roles, clear and detailed definitions of roles and responsibilities, and structured collaboration. By providing insights into these processes based on NHHAPs and field realities, and by describing seven key roles, this paper offers insights for both researchers and practitioners and will aid the development of new NHHAPs and the improvement of existing ones. Following this study, we can also suggest for future research to investigate the networks underlying and/or created through NHHAPs. For instance, what is the role of power and authority, how do relations and collaborations change over time, and what are drivers/barriers of collaboration. Besides networks and relations, other resources also impact governance and can be potential barriers for the effective implementation of NHHAPs. Future research may investigate, for instance, which resources are necessary, how they are allocated and whether there are shortages.

## Data Availability

The NHHAPs used for the desk review in this article are available online or can be obtained via the authors. The SCORCH project reports, containing more detailed information, are available online [35]. To protect the privacy and confidentiality of the research participants, the interview data cannot be shared.

## Abbreviations

HHAP: Heat health action plan
NHHAP: National heat health action plan
